# A Systematic Review of Studies Comparing Diagnostic Clinical Prediction Rules with Clinical Judgment

**DOI:** 10.1371/journal.pone.0128233

**Published:** 2015-06-03

**Authors:** Sharon Sanders, Jenny Doust, Paul Glasziou

**Affiliations:** The Centre for Research in Evidence Based Practice, Bond University, Gold Coast, 4226, Australia; University of York, UNITED KINGDOM

## Abstract

**Background:**

Diagnostic clinical prediction rules (CPRs) are developed to improve diagnosis or decrease diagnostic testing. Whether, and in what situations diagnostic CPRs improve upon clinical judgment is unclear.

**Methods and Findings:**

We searched MEDLINE, Embase and CINAHL, with supplementary citation and reference checking for studies comparing CPRs and clinical judgment against a current objective reference standard. We report 1) the proportion of study participants classified as not having disease who hence may avoid further testing and or treatment and 2) the proportion, among those classified as not having disease, who do (missed diagnoses) by both approaches. 31 studies of 13 medical conditions were included, with 46 comparisons between CPRs and clinical judgment. In 2 comparisons (4%), CPRs reduced the proportion of missed diagnoses, but this was offset by classifying a larger proportion of study participants as having disease (more false positives). In 36 comparisons (78%) the proportion of diagnoses missed by CPRs and clinical judgment was similar, and in 9 of these, the CPRs classified a larger proportion of participants as not having disease (fewer false positives). In 8 comparisons (17%) the proportion of diagnoses missed by the CPRs was greater. This was offset by classifying a smaller proportion of participants as having the disease (fewer false positives) in 2 comparisons. There were no comparisons where the CPR missed a smaller proportion of diagnoses than clinical judgment and classified more participants as not having the disease. The design of the included studies allows evaluation of CPRs when their results are applied independently of clinical judgment. The performance of CPRs, when implemented by clinicians as a support to their judgment may be different.

**Conclusions:**

In the limited studies to date, CPRs are rarely superior to clinical judgment and there is generally a trade-off between the proportion classified as not having disease and the proportion of missed diagnoses. Differences between the two methods of judgment are likely the result of different diagnostic thresholds for positivity. Which is the preferred judgment method for a particular clinical condition depends on the relative benefits and harms of true positive and false positive diagnoses.

## Introduction

Diagnostic clinical prediction rules (CPRs) are tools designed to improve clinical decision making [1]. Theoretically, CPRs, by providing objective estimates of the probability of the presence or absence of disease derived from the statistical analysis of cases with known outcomes and or by suggesting a clinical course of action, can improve the accuracy of diagnosis and or decision making.

Understanding whether and in what situations CPRs improve upon clinical judgment is an important step in the evaluation of CPRs and for the acceptance of CPRs by clinicians [2]. Existing research, which has focused on the comparative performance of CPRs and clinical judgment when both judgment methods are viewed as competing alternatives, is difficult to interpret. One body of research on the relative merits of clinical and statistical prediction has consistently reported the superior accuracy of statistical models over a clinicians ability to integrate the same data and to collect and integrate their preferred data [3–5], while another, more recent body of research has found that heuristics – proposed as models of human judgment, are on occasions more accurate than statistical models [6]. It is also difficult to know how to apply the general findings of this research to clinical practice. Many of the reviews of comparative accuracy have summarised findings from diverse professional fields including finance, medicine, psychology and education. Further, judging the clinical utility of clinical judgment and CPRs requires consideration of not just overall accuracy but the consequences of missed diagnoses (false negative) and false positive results. Results of the existing comparative research are generally not reported in a way that allows such evaluation.

We conducted a systematic review of studies that compared the performance of diagnostic CPRs with clinical judgment or the performance of the combination of CPR and clinical judgment versus either alone in the same study participants against a current and objective reference standard.

## Methods

This review was performed following methods detailed in the systematic review protocol (S1 Table– Study protocol) and is reported in line with the PRISMA Statement (S2 Table – PRISMA checklist).

### Data sources and searches

We searched MEDLINE, Embase and CINAHL from inception to January 2012, with an updated MEDLINE search to March 2013 (S3 Table – Electronic database search strategies). No limits were applied to the database searches. We also searched for systematic reviews of diagnostic CPRs using PubMed Clinical Queries. The reference lists of systematic reviews and the included studies were checked. We conducted forward searches of included studies using Science Citation Index Expanded in Web of Science and checked related citations using PubMed's Related Citations link.

### Study selection

We included studies that compared the CPRs with clinical judgment in the same participants using a current and objective reference standard. We also included studies that compared a CPR or clinical judgment alone with the combination of CPR and clinical judgment and modelling studies to determine the added value of CPRs above clinical judgment. The CPR had to have been developed using a method of statistical analysis and tested against clinical judgment in a population different (by time, location or domain) to that from which it was derived. Studies where the CPR and clinical judgment were applied to different individuals (for example, in randomised trials) or were not applied at approximately the same point in the diagnostic pathway were excluded (for example, if the result of a CPR was determined using data collected at first presentation and this was compared to clinical judgment made after further consultation, testing and observation). We excluded studies of CPRs for the diagnosis of disorders across multiple body systems, that were not applied to actual patients, that are used for the interpretation of tests such as ECGs or that are performed in selected samples of patients not consistent with populations for whom use of the CPR is not intended.

Titles and abstracts identified by the searches were screened by one reviewer and obviously irrelevant articles excluded. A second reviewer independently screened 15% of the titles and abstracts to ensure that no further studies met the inclusion criteria. After screening, potentially relevant studies were obtained in full text and independently assessed by two reviewers against the review inclusion criteria. Discrepancies were discussed and resolved with a third reviewer.

### Data extraction and risk of bias assessment

Two reviewers (SS and JD) independently extracted data on the characteristics of the study, the risk of bias and the results using a piloted data collection form. QUADAS-2 [7] was used to assess the risk of bias and concerns regarding applicability in each of the included studies. We added an additional signalling question to identify if clinical judgment and the CPR were determined independently. Discrepancies between reviewers were discussed and resolved by discussion with a third reviewer.

### Data synthesis and analysis

We grouped studies where a probability estimate, clinical diagnosis or decision was made by;

- Clinical judgment alone;
- Clinical judgment with a method of structured data collection. Clinicians may have collected data on variables contained in the CPR as per the study protocol but calculation of the results of a CPR by the clinician was not anticipated or expected, or occurred after the clinician had provided their probability estimate or diagnosis; or
- A combination of clinical judgment and clinical prediction rule, where the clinician had access to the results of the CPR but could also use their own judgment or override the CPR.

We also recorded whether the result of the CPR was calculated by the examining doctor or a researcher, the method used to elicit clinical judgment and whether clinical judgment was a clinicians probability or risk assessment (e.g. low or high risk), a diagnosis or a clinical decision.

Because many clinical prediction rules are developed to either improve the proportion of individuals with a suspected disease classified as not having the disease (thereby decreasing the number of participants undergoing further testing, referral or treatment), or to reduce the number of cases of disease missed by the current diagnostic protocol, the main outcome measures of the review were 1) the percent of study participants classified as not having the disease by the CPR or clinical judgment ((False negative (FN)+ True negative (TN))/total number of participants in the study (total N). The higher this proportion, the fewer individuals that may undergo further testing, referral and or treatment, and 2) the percent of study participants among those classified by the CPR or clinical judgment as not having the disease who actually have the disease (FN/(FN+TN) or 1-negative predictive value). It is desirable that this be as close to 0% as possible. We also report measures of diagnostic accuracy including the sensitivity (True positive (TP)/(TP+ FN)) and 4) specificity (True negative (TN)/(FP+TN)) of CPRs and clinical judgment, and present graphically the proportion of all study participants who are classified by CPRs and clinical judgment as having disease who do (True Positives/total N) and do not (False Positives/total N) and the proportion of all participants who are classified as not having disease who do (False Negatives/total N) and do not (True Negatives/total N).

We did not perform a meta-analysis due to clinical and statistical heterogeneity. Instead, we synthesised the results of the included studies overall, and by clinical condition (where there were 2 or more studies available) by determining the number of comparisons in which the proportion of participants classified as not having disease and the proportion of missed cases of disease (missed diagnoses) in participants classified as not having disease for CPRs and clinical judgement was similar, greater or lesser. To determine whether there was a difference in the proportion classified as not having disease between CPRs and clinical judgment we conducted a statistical test of the difference between two proportions from dependent samples. To obtain the statistical significance of the relative difference in the proportion classified by CPRs and clinical judgment as not having disease that do, we conducted a test of the strength of association between two proportions (false negative rates) from dependent samples. If studies reported different thresholds for clinical judgment or the CPR, and if the proportions (i.e. those classified as not having disease and the proportion of missed diagnoses) were similar at the different thresholds (this only occurred in 1 study included in this review) we reported only the comparison for the threshold with the highest Youden’s index ((sensitivity + specificity)-1).

## Results

### Literature search

**Fig 1:**
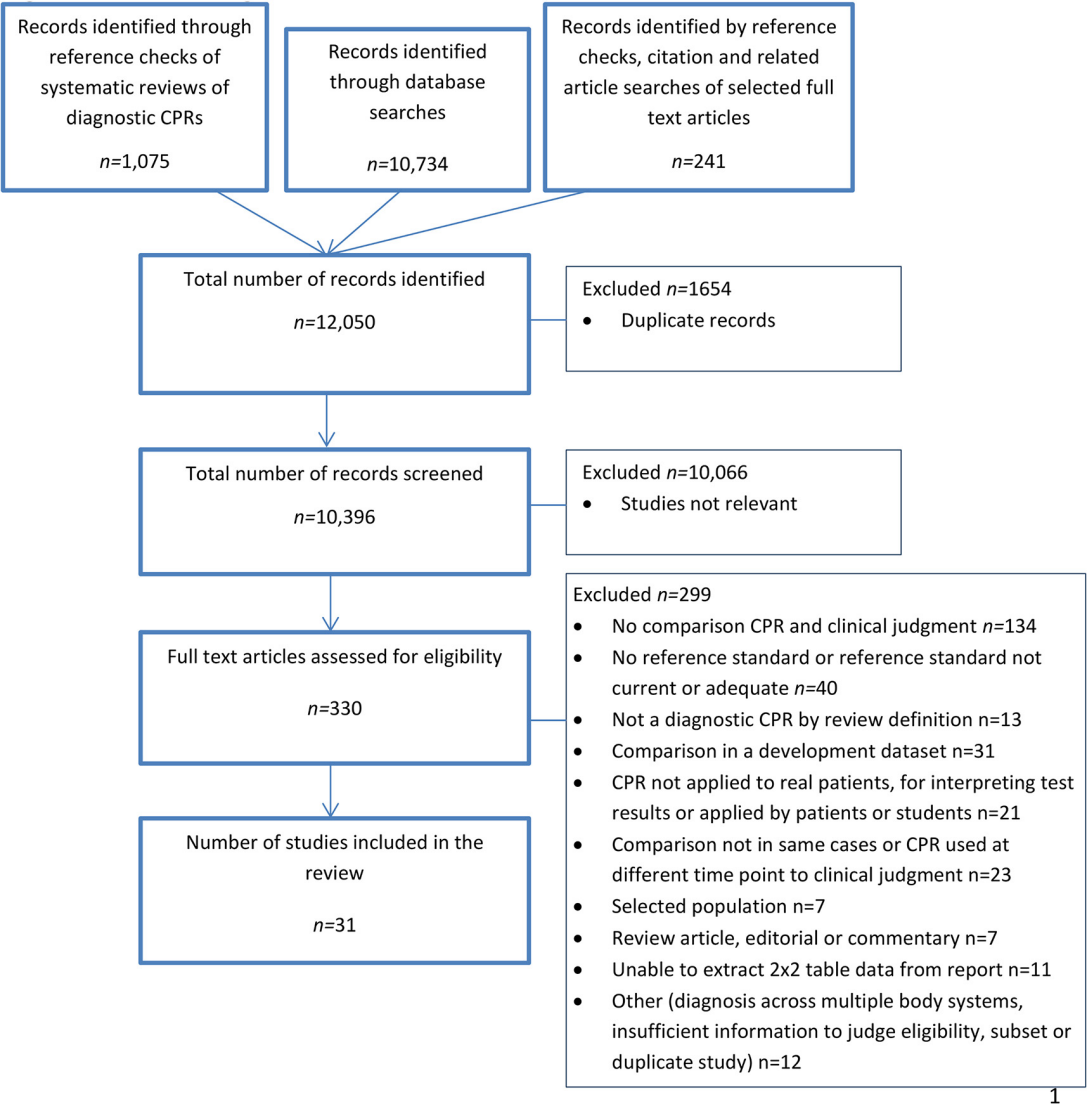
PRISMA flow diagram of article selection process.

Of 10,155 titles and abstracts screened against review eligibility criteria, 330 were obtained in full text and assessed for eligibility by two reviewers. 31 studies [8–38] were included in the review (Fig 1 – PRISMA flow diagram of article selection process).

### Study characteristics

The studies addressed a variety of conditions: 9 for pulmonary embolism (PE) [8–16], 6 for deep vein thrombosis (DVT) [17–22], 3 for streptococcal throat infection [23–25], 3 for ankle and/or foot fracture [26–28], 2 for acute appendicitis [29, 30] and one each for acute coronary syndrome [31], pneumonia [32], head injury in children [33], cervical spine injury [34], active pulmonary tuberculosis [35], malaria [36], bacteraemia [37] and influenza [38] (Table 1 – Clinical conditions and study comparisons). Twenty five different CPRs were evaluated. The majority (n = 16) were derived from logistic regression analysis and the remainder from recursive partitioning analysis (n = 3), discriminant analysis (n = 2), neural networking (n = 1), simple Bayesian analysis (n = 2) and an unspecified multivariable analysis (n = 1). In just over half of the included studies (n = 17), clinical judgment was a clinicians estimate of the probability of the presence of disease or categorisation of a study participant into a risk group (e.g. low, intermediate or high risk). In the remaining studies, clinical judgment was a clinicians diagnosis (n = 8), intended management (n = 3) or the clinical action taken (n = 3). In half of the included studies (n = 15) the experience of clinicians estimating the probability of the target disorder or making a diagnosis or management decision was not reported. Ten studies included clinicians with varying levels of experience (e.g. ‘post graduates’ and ‘confirmed emergency physicians’), 3 included specialists only and 3 junior staff only.

**Table 1:** Clinical conditions and study comparisons.

Clinical condition	Number of studies (number of comparisons)	Methods of estimating a probability, making a diagnosis or management decision being compared (number of comparisons)
Pulmonary embolism [8–16]	9* (16)	CPR versus clinical judgment alone (1)
		CPR versus clinical judgment + structured data collection (13)
		CPR versus combination of clinical judgment and CPR (2)
Deep vein thrombosis [17–22]	6 (7)	CPR versus clinical judgment alone (1)
		CPR versus clinical judgment + structured data collection (5)
		CPR versus combination of clinical judgment and CPR (1)
Streptococcal throat infection [23–25]	3 (5)	CPR versus clinical judgment + structured data collection (5)
Ankle or foot fracture [26–28]	3 (4)	CPR versus clinical judgment alone (1)
		CPR versus clinical judgment + structured data collection (3)
Acute appendicitis [29–30]	2 (2)	CPR versus clinical judgment alone (1)
		CPR versus combination of clinical judgment and CPR (1)
Acute coronary syndrome [31]	1 (1)	CPR versus clinical judgment + structured data collection (1)
Pneumonia [32]	1 (4)	CPR versus clinical judgment + structured data collection (4)
Abnormalities on computed tomography scan in child with head injury [33]	1 (1)	CPR versus clinical judgment alone (1)
Cervical spine injuries [34]	1 (1)	CPR versus combination of clinical judgment and CPR (1)
Active pulmonary tuberculosis [35]	1 (1)	CPR versus clinical judgment + structured data collection (1)
Malaria [36]	1 (2)	CPR versus clinical judgment alone (2)
Bacteremia [37]	1 (1)	CPR versus clinical judgment + structured data collection (1)
Influenza [38]	1 (1)	CPR versus clinical judgment alone (1)

* 8 cohorts

### Risk of bias

**Fig 2:**
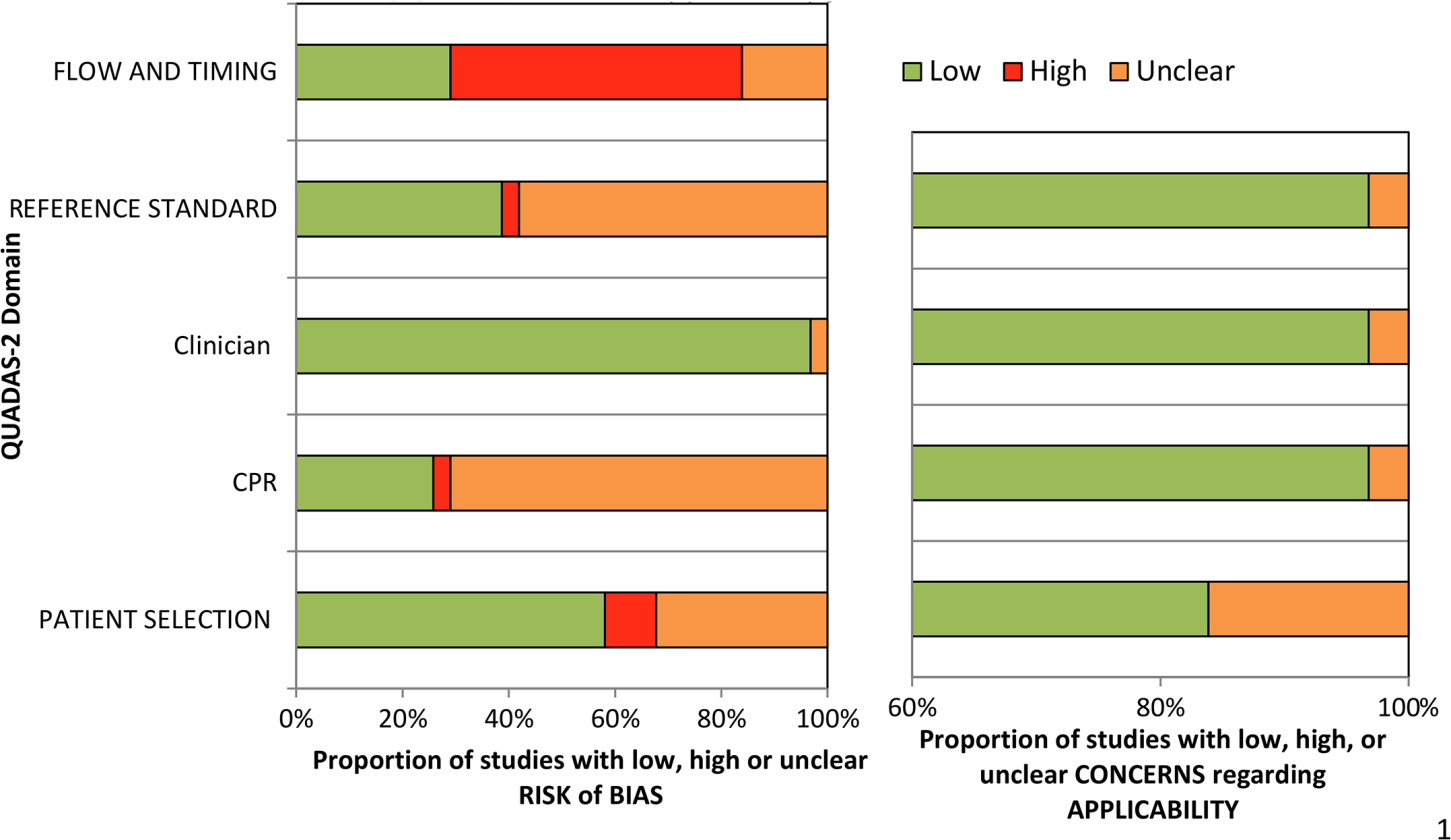
Summary QUADAS-2 [7] risk of bias and applicability judgments.

87% (27/31) of studies were judged to be at high or unclear risk of bias on two or more domains of the QUADAS-2 tool (Fig 2 – Summary QUADAS-2 risk of bias and applicability judgments). The most common risk of bias was due to interpretation of the reference standard occurring with knowledge of the index test result. For most studies in which the CPR was applied retrospectively to the data, it was not possible to determine whether researchers were blind to the result of the reference standard test. This is likely to bias results in favour of the CPR. 55% (17/31) of studies were judged to be at high risk of bias on the flow and timing domain. Studies commonly failed to include all enrolled cases in the data analysis or incorporated one of the index tests in the reference standard. Risks of bias assessments for individual studies are shown in Table 2 – Risk of bias and applicability concerns for individual studies included in the review.

**Table 2:** Risk of bias and applicability concerns for individual studies included in the review.

Study	Risk of Bias					Concerns regarding Applicability			
	Patient selection	Index test (CPR)^*^	Index test (clinical judgment)	Reference standard^†^	Flow and timing ^‡^	Patient selection	Index test (CPR)	Index test (clinical judgment)	Reference standard
Pulmonary embolism									
Runyon et al, 2005 [8]	Unclear	Unclear	Low	Low	High	Low	Low	Low	Low
Kabrhel et al, 2009 [9]	Low	Unclear	Low	Unclear	High	Unclear	Low	Low	Low
Kline et al, 2008 [10]	Low	Unclear	Low	Unclear	Unclear	Unclear	Low	Low	Low
Kabrhel et al, 2005[11]	Unclear	Unclear	Low	Unclear	High	Unclear	Low	Low	Low
Carrier et al, 2006 [12]	Low	Unclear	Low	Unclear	High	Low	Low	Low	Low
Chagnon et al, 2002 [13]	Low	Unclear	Low	Unclear	High	Unclear	Low	Low	Low
Penaloza et al, 2012 [14]	Low	Unclear	Low	Unclear	High	Low	Low	Low	Low
Sanson et al, 2000 [15]	Low	Unclear	Low	Unclear	High	Low	Low	Low	Low
Penaloza et al, 2013 [16]	Low	Unclear	Low	Unclear	High	Low	Low	Low	Low
Deep vein thrombosis									
Geersing et al, 2010 [17]	Low	Low	Low	Unclear	High	Low	Low	Low	Low
Bigaroni et al,2000[18]	Low	Low	Low	Unclear	Unclear	Low	Low	Low	Low
Miron et al, 2000 [19]	Low	Low	Low	High	High	Low	Low	Low	Low
Blattler et al, 2004 [20]	Low	High	Low	Unclear	Low	Low	Low	Low	Low
Cornuz et al, 2002 [21]	Low	Low	Low	Low	High	Low	Low	Low	Low
Wang et al, 2013 [22]	Unclear	Unclear	Low	Unclear	Unclear	Low	Low	Low	Low
Streptococcal throat infection									
Cebul and Poses, 1986 [23]	Unclear	Unclear	Low	Low	High	Low	Low	Low	Low
Rosenberg et al, 2002 [24]	High	Unclear	Low	Low	High	Low	Low	Low	Low
Attia et al, 2001 [25]	Unclear	Unclear	Low	Low	High	Unclear	Low	Low	Low
Ankle or foot fracture									
Glas et al, 2002 [26]	Low	Unclear	Low	Low	Low	Low	Low	Low	Low
Singh-Ranger and Marathias, 1999 [27]	Low	Low	Low	Unclear	Low	Low	Low	Low	Low
Al Omar and Baldwin, 2002 [28]	Unclear	Low	Low	Unclear	Low	Low	Low	Low	Low
Conditions with ≤ 2 studies									
Fenyo, 1987 [29]	Low	Low	Low	Unclear	High	Low	Low	Low	Low
Meltzer et al, 2013 [30]	Unclear	Unclear	Low	Unclear	High	Low	Low	Low	Low
Mitchell et al, 2006 [31]	Low	Unclear	Low	Unclear	Unclear	Low	Low	Low	Low
Emerman et al, 1991 [32]	Unclear	Unclear	Low	Low	Low	Low	Low	Low	Low
Crowe et al, 2010 [33]	High	Unclear	Low	Unclear	Unclear	Low	Unclear	Low	Low
Vaillancourt et al, 2009 [34]	High	Unclear	Low	Low	High	Low	Low	Low	Low
El-Solh et al, 1999 [35]	Low	Unclear	Low	Low	Low	Low	Low	Low	Unclear
Bojang et al, 2000 [36]	Unclear	Low	Low	Low	Low	Low	Low	Low	Low
Leibovici et al, 1991 [37]	Low	Unclear	Unclear	Low	Low	Low	Low	Low	Low
Stein et al, 2005[38]	Unclear	Unclear	Low	Low	Unclear	Low	Low	Low	Low

* In studies where the CPR is applied retrospectively to the data by the researcher using predictor data collected by the clinician, if there was no statement that researchers were blind to the reference standard the risk of bias was considered to be unclear. If predictor data was collected by the researcher and there was no statement that researchers were blind to the reference standard, the risk of bias was considered to be high.

†When the reference standard comprised subjective tests, if there was no statement that those interpreting the reference standard tests were blind to the results of either the CPR or clinician, the risk of bias was considered to be unclear.

‡If the method of determining disease status involved a combination of different tests in which some tests were applied to some patients and one test applied to all patients (differential verification) then the risk of bias was considered to be unclear. If performance of any of the reference standard tests was dependent upon the results of the index test, the risk of bias was considered to be high. If it was not possible to determine whether all eligible patients had been included in the analysis the risk of bias was considered to be unclear. If it was clear that not all patients had been included in the analysis (due to missing outcome data or because data from the clinicians estimate or data necessary to derive the results of the CPR were not available) and these studies reported results for the comparisons in different numbers of cases or only presented the results for cases on which data for both the comparisons was available, the risk of bias was considered to be high. Risk of bias was recorded as high if either of the issues relating to the reference standard test or analysis were high.

### Study results

**Fig 3:**
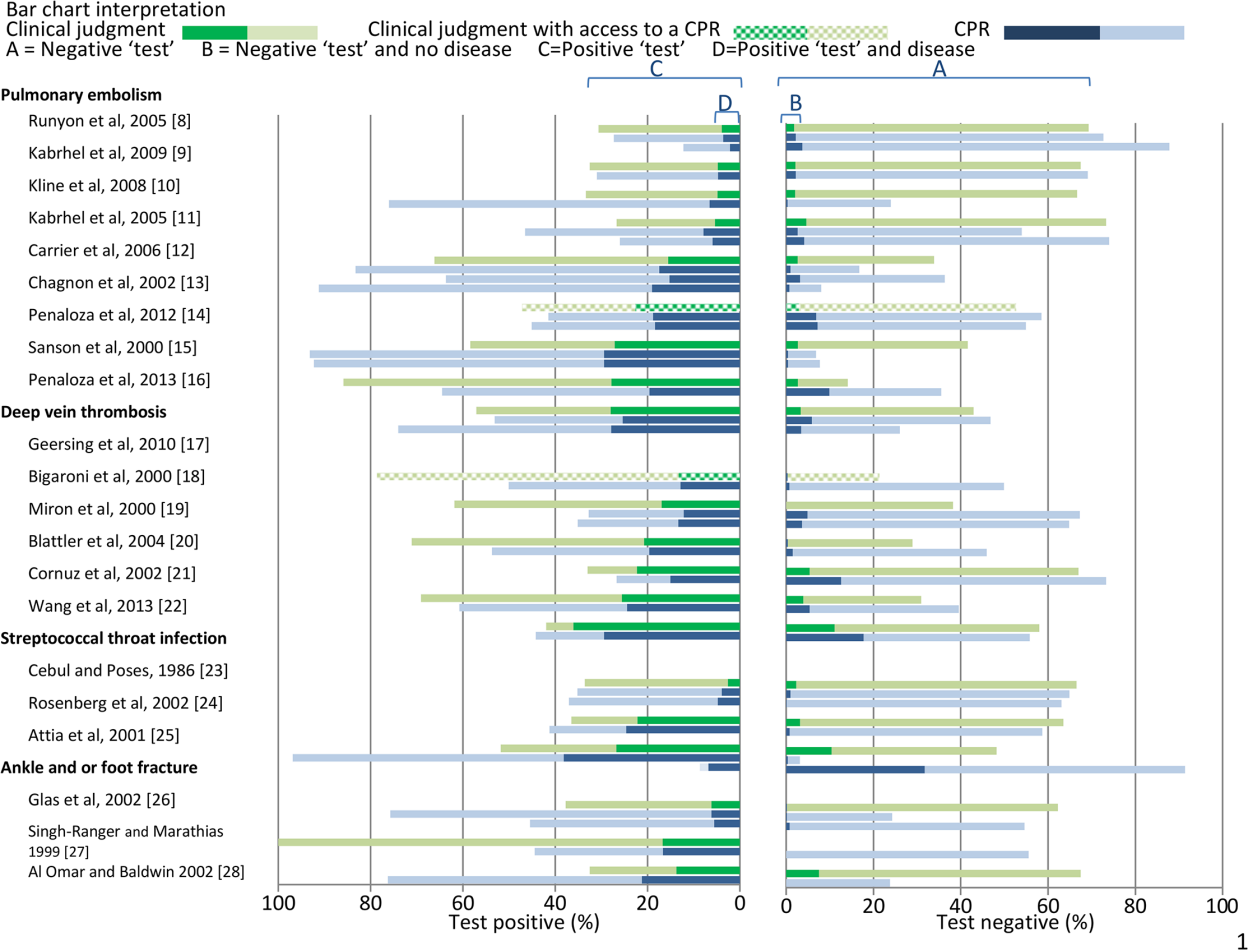
Results of the included studies.

**Fig 4:**
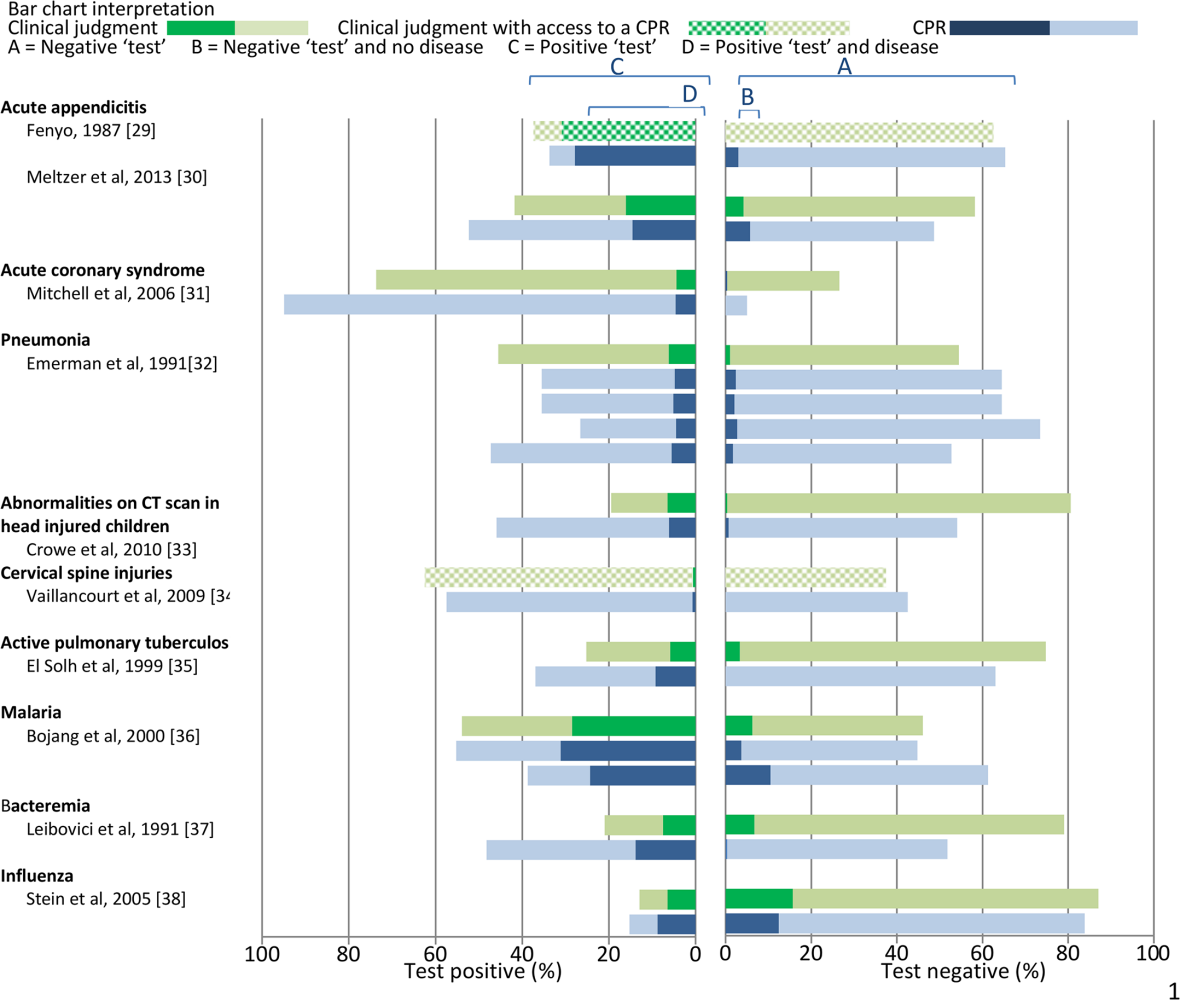
Results of the included studies for conditions with ≤ 2 studies.

Results of the included studies are Tabulated in Table 3 – Characteristics and results of included studies, Table 4 – Characteristics and results of included studies for conditions with ≤ 2 studies, and presented graphically in Fig 3 – Accuracy estimates of clinical judgment versus CPRs for the included studies and Fig 4 – Accuracy estimates of clinical judgment versus CPRs for the included studies for conditions with ≤2 studies.

**Table 3:** Characteristics and results of included studies.

Study	Setting	Method of establishing status of target disorder	Prevalence (n/N)	Comparison (method of estimating the probability of target disorder, making a diagnosis or management decision)	Threshold (low risk if)	Sensitivity (95% CI)	Specificity (95% CI)	% missed cases of disease among those classified as not having disease (95% CI)*	% classified as not having disease (95% CI)^+^
**Pulmonary embolism**									
Runyon et al, 2005 [8]	ED	Medical record review, F/U by mail or telephone and death records at 1.5 months	6% (144/2477)	**Clinical judgment** + structured data collection	<15%	69 (61–76)	72 (70–74)	2.6 (1.9–3.5)	69 (67–71)
				**Wells PE score** calculated by researcher	<2	62 (54–70)	75 (73–77)	3.0 (2.3–3.9)	73 (70–74)
				**Charlotte score** calculated by clinician	Safe	36 (28–45)	89 (88–91)	4.2 (3.5–5.2)	88 (87–89)
Kabrhel et al, 2009 [9]	ED	Adjudicated review of imaging results, medical records and F/U at 1.5 months	7% (545/7940)	**Clinical judgment** alone	<15%	69 (65–73)	70 (69–71)	3.1 (2.7–3.6)	68 (67–69)
				**Wells PE score** calculated by researcher	<2	68 (64–72)	72 (71–73)	3.2 (2.7–3.7)	69 (68–70)
Kline et al, 2008 [10]	ED	Adjudicated review of imaging results, medical records and F/U at 1.5 months	7% (561/8138)	**Clinical judgment** + structured data collection	<15%	71 (67–74)	69 (68–71)	3.0 (2.6–3.5)	67 (66–68)
				**PERC Rule** calculated by researcher	No criteria present	96 (94–97)	25 (24–26)	1.3 (0.9–1.9)	24 (23–25)
Kabrhel et al, 2005 [11]	ED	Review of medical records at 3 months F/U	10% (61/607)	**Clinical judgment** + structured data collection	Alternate diagnosis not less likely	54 (41–67)	76 (73–80)	6.3 (4.4–8.9)	73 (70–77)
				**Wells PE score** calculated by researcher	<2	79 (66–88)	57 (53–61)	4.0 (2.4–6.7)	54 (50–58)
					≤4	59 (46–72)	78 (74–81)	5.5 (3.8–8.1)	74 (70–77)
Carrier et al, 2006 [12]	NMD	Patient follow-up by telephone or return appointment at 3 months	18% (76/413)	**Clinical judgment** + structured data collection	<20%	86 (76–93)	38 (33–43)	7.5 (4.3–13.0)	34 (30–38)
				**Wells PE score** calculated by researcher	≤2	95 (87–99)	19 (15–24)	5.8 (2.3–14.0)	17 (13–21)
					≤4	83 (73–91)	41 (35–46)	8.7 (5.1–14.3)	36 (32–41)
				**Rodgers model** calculated by researcher	No criteria present	96 (89–99)	9 (6–13)	3.2 (1.1–8.9)	24 (20–28)
Chagnon et al, 2002 [13]	ED	Follow-up (method not specified) at 3 months	26% (71/277)	**Clinical judgment +** access to the **Geneva Score**	‘low’	89 (79–95)	67 (60–73)	5.5 (2.8–10.4)	53 (47–59)
				**Wells PE score** calculated by researcher	<2	73 (61–83)	69 (63–76)	11.7 (7.6–17.6)	59 (53–64)
				**Geneva Score** calculated by clinician	≤4	72 (60–82)	64 (57–71)	13.2 (8.7–19.5)	55 (49–61)
Penaloza et al, 2012 [14]	ED	Review of imaging results, medical records and patient or relative follow-up at 3 months	30% (286/959)	**Clinical judgment** + structured data collection	‘low’	91 (87–94)	55 (52–59)	6.5 (4.5–9.4)	42 (39–45)
				**Revised Geneva Score + PERC Rule** calculated by researcher	<4 and no criteria present	99 (97–99.6)	9 (7–12)	6.2 (2.4–14.8)	7 (5–9)
				**PERC Rule** calculated by researcher	No criteria present	99 (97–99.6)	10 (8–13)	5.4 (2.1–13.1)	8 (6–10)
Sanson et al, 2000 [15]	IP, OPD	Perfusion lung scintigraphy or pulmonary angiography	31% (160/517)	**Clinical judgment** + structured data collection	<20%	91 (85–96)	16 (12–21)	19.0 (10.9–30.9)	14 (11–18)
				**Wells PE score** calculated by researcher	<2	66 (57–75)	36 (31–42)	27.9 (21.3–35.6)	36 (31–40)
Penaloza et al, 2013 [16]	ED	Review of imaging results, medical records and patient or relative follow-up at 3 months	31% (325/1038)	**Clinical judgment + s**tructured data collection	‘low’	90 (86–93)	58 (54–61)	7.6 (5.5–10.5)	43 (40–46)
				**Wells PE score** calculated by researcher	<2	82 (77–85)	60 (56–63)	12.6 (9.9–15.8)	47 (44–50)
				**Revised Geneva Score** calculated by researcher	<4	89 (85–92)	33 (30–37)	13.0 (9.5–17.5)	26 (23–29)
**Deep vein thrombosis**									
**Study**	**Setting**	**Method of establishing status of target disorder**	**Prevalence (n/N)**	**Comparison (method of estimating the probability of target disorder, making diagnosis or management decision)**	**Threshold (low risk if)**	**Sensitivity (95% CI)**	**Specificity (95% CI)**	**% missed cases of disease among those classified as not having disease (95% CI)**	**% classified as not having disease (95% CI)**
Geersing et al, 2010 [17]	PC	Clinical probability, ultrasound and F/U at 3 months	14% 136/1002	**Clinical judgment +** access to the **Oudega Rule**	<10%	98 (94–99)	24 (22–27)	1.4 (0.5–4.0)	21 (19–24)
				**Oudega Rule** calculated by clinician	< = 3	95 (90–98)	57 (54–60)	1.4 (0.6–2.9)	50 (47–53)
Bigaroni et al, 2000 [18]	ED, OPD	D-dimer, ultrasound, other imaging and telephone F/U at 3 months	17% (28/165)	**Clinical judgment** +structured data collection	‘Low risk’	98 (85–99.8)	46 (38–54)	0.0 (0.0–5.8)	38 (31–46)
				**Wells DVT Score** calculated by junior clinician	<1	71 (53–85)	75 (67–82)	7.2 (3.7–13.6)	67 (60–74)
				**Wells DVT score** calculated by senior clinician	<1	79 (61–90)	74 (66–80)	5.6 (2.6–11.7)	65 (57–72)
Miron et al, 2000 [19]	ED, OPD	D-dimer, ultrasound, other imaging and telephone F/U at 3 months	21%(57/270)	**Clinical judgment** + structured data collection	<20%	98 (91–99.7)	36 (30–43)	1.3 (0.2–6.9)	29 (24–35)
				**Wells DVT score** calculated by researcher	<1	93 (83–97)	57 (50–63)	3.2 (1.3–7.9)	46 (40–52)
Blattler et al, 2004 [20]	OPD	Ultrasound and telephone F/U at 6+ months	28%(57/206)	**Clinical judgment** + structured data collection (includes D-dimer)	‘Low risk’	81(69–89)	85 (79–90)	8.0 (4.5–13.7)	67 (60–73)
				**Wells DVT score** calculated by researcher	Low	54 (42–67)	84 (77–89)	17.2 (12.0–24.0)	73 (67–79)
Cornuz et al, 2002 [21]	VL	Ultrasound, other imaging, mail or telephone F/U	29% (82/278)	**Clinical judgment** + structured data collection	<20%	87 (78–92)	38 (32–45)	12.8 (7.3–21.5)	31 (26–37)
				**Wells DVT score** calculated by researcher	<1	83 (73–90)	49 (42–56)	12.8 (7.8–20.4)	39 (34–45)
Wang et al, 2013 [22]	OPD	Ultrasound and telephone or email F/U at 1.5 months	47% (191/405)	**Clinical judgment** alone	‘Safe’	76 (70–82)	89 (84–92)	19.2 (14.6–24.7)	58 (53–63)
				**Wells DVT score** ** calculated by clinician	< = 1	62 (55–69)	72 (66–78)	31.9 (26.1–38.2)	56 (51–61)
**Study**	**Setting**	**Method of establishing status of target disorder**	**Prevalence (n/N)**	**Comparison (method of estimating the probability of target disorder, making diagnosis or management decision)**	**Threshold (low risk if)**	**Sensitivity (95% CI)**	**Specificity (95% CI)**	**% missed cases of disease among those classified as not having disease (95% CI)**	**% classified as not having disease (95% CI)**
**Streptococcal throat infection**									
Cebul and Poses, 1986 [23]	PC/ Adults	Throat culture	5% (15/310)	**Clinical judgment** + structured data collection	No treatment	53 (27–79)	68 (62–73)	3.4 (1.7–6.9)	67 (61–72)
				**Walsh model +Tomkins management rule** calculated by researcher	No treatment	80 (52–95)	67 (61–72)	1.5 (0.5–4.3)	65 (59–70)
				**Centor model + Tomkins management rule** calculated by researcher	No treatment	100 (75–100)	66 (60–72)	0.0 (0.0–2.2)	63 (57–69)
Rosenberg et al, 2002 [24]	ED/ Mixed	Pharyngeal swab culture	25% (32/126)	**Clinical judgment** + structured data collection + rapid test	No treatment	90 (74–98)	92 (87–95)	5.0 (2.0–12.2)	64 (55–71)
				**Modified Centor Score** calculated by researcher	No treatment	97 (84–99.5)	78 (68–86)	1.4 (0.2–7.3)	59 (50–67)
Attia et al, 2001 [25]	ED, OPD/Children	Tonsillopharyngeal swab culture	37% (218/587)	**Clinical judgment** + structured data collection	< = 50%	72 (66–78)	60 (55–65)	21.6 (17.2–26.7)	48 (44–52)
				**Clinical prediction rule of Attia** calculated by researcher	0	99 (97–99.9)	5 (3–7)	11.8 (3.3–34.3)	3 (2–5)
					< = 3	18 (13–24)	97 (95–99)	34.7 (30.7–39.0)	91 (89–94)
**Ankle and or foot fracture**									
**Study**	**Setting**	**Method of establishing status of target disorder**	**Prevalence (n/N)**	**Comparison (method of estimating the probability of target disorder, making diagnosis or management decision)**	**Threshold (low risk if)**	**Sensitivity (95% CI)**	**Specificity (95% CI)**	**% missed cases of disease among those classified as not having disease (95% CI)**	**% classified as not having disease (95% CI)**
Glas et al, 2002 [26]	ED/ Adults	Ankle and midfoot x-ray	6% (41/647)§	**Clinical judgment** + structured data collection	No X-ray	98 (87–99.6)	66 (62–70)	0.3 (0.0–1.4)	62 (59–66)
				**OAR – ankle and foot** calculated by researcher	Negative	98 (87–99.6)	26 (22–29)	0.6 (0.1–3.5)	24 (21–28)
				**Leiden ankle rule** calculated by researcher	< = 7	88 (74–96)	57 (53–61)	1.4 (0.6–3.3)	55 (51–58)
Singh-Ranger and Marathias 1999 [27]	ED/ Adults	Ankle x-ray	17% (3/18)ǁ	**Clinical judgment** alone	No fracture	100 (31–100)	0.0 (0.0–22)	0.0 (0.0–0.0)	0 (0–18)
				**OAR – ankle** calculated by researcher	Negative	100 (31–100)	67 (38–85)	0.0 (0.0–27.8)	56 (34–75)
Al Omar and Baldwin 2002 [28]	ED/ Children	Ankle or midfoot x-ray	21% (17/80) §¶	**Clinical judgment** + structured data collection	No fracture	65 (38–86)	76 (64–86)	11.1 (5.2–22.2)	68 (57–77)
				**OAR – ankle and foot** calculated by researcher	Negative	100 (80–100)	30 (19–43)	0.0 (0.0–16.8)	24 (16–34)

*% missed cases of disease among those classified as not having disease (FN/FN+TN or 1-negative predictive value)

†% classified as not having disease (FN+TN/total study N)

§ankle or midfoot fracture

ǁ ankle fracture Includes Salter Harris fractures

**2-category Wells DVT score

ED – emergency department OPD – Outpatient department VL – vascular laboratory PC – primary care NMD – nuclear medicine department F/U – follow-up OAR – Ottawa ankle rules PE – pulmonary embolism DVT- deep vein thrombosis

**Table 4:** Characteristics and results of included studies for conditions with ≤2 studies.

Study	Setting	Method of establishing disease status	Prevalence (n/N)	Comparison (method of estimating probability, making diagnosis or management decision)	Threshold (low risk if)	Sensitivity (95%CI)	Specificity (95% CI)	% missed cases of disease among those classified as not having disease (95% CI)	% classified as not having disease (95% CI)
Fenyo, 1987 [29]	IP	Intraop diagnosis, histopathology of excised appendices and record review at 1–2 years	31% (256/830)	**Clinical judgment** + access to results of **Fenyo Score**	No surgery	100 (99–100)	91 (88–93)	0.0 (0.0–0.7)	63 (59–66)
				**Fenyo Score** calculated by researcher	≤11	90 (86–94)	92 (89–94)	4.6 (3.1–6.6)	66 (63–69)
Meltzer et al, 2013 [30]	ED	Surgical pathology, CT scan or telephone F/U at 7 days	20% (53/261)	**Clinical judgment alone**	Appendicitis not most likely diagnosis	79 (66–89)	68 (61–74)	7.2 (4.1–12.5)	58 (52–64)
				**Modified Alvarado score** calculated by researcher	<4	72 (58–83)	54 (47–61)	11.8 (7.3–18.6)	49 (43–55)
Mitchell et al, 2006[(31]	ED	Review of medical records and telephone F/U at 1.5 months	5% (51/1114)	**Clinical judgment** + structured data collection	≤2%	96 (87–99)	27 (25–30)	0.7 (0.2–2.5)	26 (24–29)
				**ACI-TIPI** calculated by researcher	≤2%	100 (93–100)	5 (4–7)	0.0 (0.0–6.4)	5 (4–7)
Emerman et al, 1991 [32]	ED, OPC	Posteroanterior and lateral chest x-ray	7% (21/290)	**Clinical judgment** + structured data collection	No radiograph	86 (64–97)	58 (52–64)	1.9 (0.7–5.4)	55 (49–60)
				**Diehr Score** calculated by researcher	≤0	67 (43–85)	67 (61–73)	3.7 (1.8–7.5)	65 (59–70)
				**Heckerling Score** calculated by researcher	<2	71 (48–89)	67 (61–73)	3.3 (1.5–6.8)	65 (59–70)
				**Gennis Rule** calculated by researcher	No variable present	62 (39–82)	76 (70–81)	3.8 (1.9–7.2)	74 (68–78)
				**Singal Score** calculated by researcher	Probability <0.26	76 (53–92)	55 (49–61)	3.3 (1.4–7.4)	53 (47–58)
Crowe et al, 2010 [33]	ED	Medical record review of imaging tests, observation and readmission	7% (73/1065)	**Clinical judgment alone**	No CT scan	95 (87–98)	86 (84–88)	0.5 (0.2–1.2)	81 (78–83)
				**CHALICE criteria** calculated by researcher	No criteria present	89 (80–95)	57 (54–60)	1.4 (0.7–2.7)	54 (51–57)
Vaillancourt et al, 2009 [34]	ES	Radiographic imaging and telephone or mail F/U at 14 days	1% (12/1974)	**Clinical judgment** + access to results of **Canadian C-Spine Rule**	Negative	100 (73–100)	38 (36–40)	0.0 (0.0–0.5)	38 (35–40)
				**Canadian C-Spine Rule** score calculated by researcher	Negative	100 (73–100)	43 (40–45)	0.0 (0.0–0.6)	43 (40–45)
El Solh et al, 1999 [35]	IP	Culture of respiratory specimens	9% (11/119)	**Clinical judgment** + structured data collection	No active TB	64 (31–89)	79 (70–86)	4.5 (1.8–11.0)	75 (66–82)
				**El Sohl** rule calculated by researcher	Negative	100 (71–100)	69 (60–78)	0.0 (0.0–4.9)	63 (54–71)
Bojang et al, 2000 [36]	OPD	Temperature and parasitemia on blood film	35% (133/382)	**Clinical judgment alone**	No malaria	82 (74–88)	61 (55–67)	13.6 (9.3–19.5)	46 (41–51)
				**Olaleye algorithm** calculated by researcher	<7	90 (83–94)	63 (57–69)	8.2 (4.9–13.3)	46 (41–51)
					<8	90 (83–95)	78 (72–83)	17.1 (12.8–22.4)	61 (56–66)
Leibovici et al, 1991[37]	IP	Blood culture	14% (36/257)	**Clinical judgment** + structured data collection	No bacteremia	53 (36–70)	84 (79–89)	8.5 (5.4–13.2)	79 (74–84)
				**Rule of Leibovici** calculated by researcher	<20%	97 (85–99.5)	60 (53–67)	0.8 (0.1–4.2)	52 (46–58)
Stein et al, 2005 [38]	ED	Reverse transcriptase PCR assay for influenza A and B	21% (53/258)	**Clinical judgment alone**	No influenza	29 (17–44)	92 (87–95)	18.0 (13.2–24.1)	87 (82–91)
				**Cough and fever rule** calculated by researcher	Negative	41 (27–57)	92 (87–95)	14.8 (10.4–20.7)	84 (78–88)

*% missed cases of disease (FN/FN+TN or 1-NPV)

†% classified as low risk (FN+TN/total N)

There were 41 comparisons between CPRs and clinical judgment [8–12, 14–16, 18–28, 30–33, 35–38] (Table 3, Table 4, Fig 3 and Fig 4). In 2 (5%) comparisons (10, 37), CPRs reduced the proportion of missed diagnoses in those classified as not having the disease, but this was offset by classifying a larger proportion of study participants as having disease (more false positives). In 33 (80%) comparisons [8, 9, 11, 12, 14, 15, 18, 19, 21, 23– 28, 30– 33, 35, 36, 38] the proportion of diagnoses missed by the CPR and clinical judgment was similar and in 7 of these comparisons [15, 18, 19, 27, 32, 36] CPRs classified a larger proportion of participants as not having disease (fewer false positives) and a similar proportion in 16 [8, 9, 11, 12, 21, 23–26, 30, 32, 35, 36, 38]. In 6 (15%) comparisons [8, 16, 20, 22, 25] the proportion of diagnoses missed by the CPR was greater. This was offset by classifying a smaller proportion of participants as having the disease (fewer false positives) in 2 [8, 25] comparisons. In 3 of the 6 comparisons [16, 20, 22] the CPRs classified a similar proportion of participants as having the disease. There was 1 comparison [16] where the CPR both missed more diagnoses and classified a larger proportion of participants as having the disease (more false positives), but no comparisons where the CPR missed fewer diagnoses and classified a larger proportion of participants as not having disease.

There were 5 comparisons between CPRs and the combination of CPR and clinical judgment [13, 17, 29, 34] (Table 3, Table 4, Fig 3 and Fig 4). In 3 (60%) comparisons the proportion of diagnoses missed was similar [13, 17, 34] and in 2 [17, 34] of these comparisons, CPRs classified a larger proportion of study participants as not having disease (fewer false positives) than the combination of CPR and clinical judgment. In 2 (40%) comparisons [13, 29], the proportion of diagnoses missed by the CPRs was greater while the proportion classified as not having disease by the CPRs and the combination of CPR and clinical judgment was similar. There were no comparisons between the combination of CPR and clinical judgment and clinical judgment alone.

There were 5 studies [11, 12, 17, 25, 36] of 10 comparisons, that used different thresholds for the CPR or clinical judgment (for example, Kabrhel et al, 2005 [11] compared clinical judgment to the Wells PE score at threshold <2 and ≤4). We report on the results of 9 of these comparisons, excluding the results of 1 comparison [17] where the proportions of interest (that is, the proportion classified as having disease or the proportion of missed diagnoses) were similar at the different thresholds. This means that for a small number of comparisons (n = 4) clinical judgment is counted twice [11, 12, 25, 36].

### Pulmonary embolism

From 9 studies in pulmonary embolism, there were 9 comparisons between the Wells PE score (original 3 level or 2 level score) and clinical judgment [8, 9, 11, 12, 13, 15, 16]. In 8 (89%) comparisons [8, 9, 11–13, 15], the proportion of diagnoses missed by the score and clinical judgment was similar. In 1 of these [15], the score classified a larger proportion of all participants as not having the disease (fewer false positives), a similar proportion in 5 comparisons [8, 9, 11, 12, 13] and a larger proportion of participants as having the disease (more false positives) in 2 [11, 12]. In 1 (11%) comparison [16], the proportion of diagnoses missed by the Wells PE score was greater, while the proportion of participants classified as not having the disease was similar. In 2 comparisons between the PERC Rule and clinical judgment [10, 14], the rule reduced the proportion of missed diagnosis in 1 [10], but this was offset by classifying a larger proportion of participants as having the disease (more false positives). In the other comparison [14], the proportion of diagnoses missed by the PERC rule and clinical judgment was similar. In 1 comparison [16] the Revised Geneva Score both missed more diagnoses and classified a larger proportion of participants as having the disease than clinical judgment. In 1 comparison [13] between the Geneva score and the combination of clinical judgment and score, the proportion of diagnoses missed by the CPR was greater.

### Deep vein thrombosis

From 6 studies of DVT, there were 6 comparisons between the Wells DVT score and clinical judgment [18–22]. There were no comparisons in which the score reduced the proportion of missed diagnoses. In 4 (67%) comparisons the proportion of diagnoses missed by the score and clinical judgment was similar [18, 19, 21]. In 3 of these [18, 19] the score classified a larger proportion of all participants as not having disease (fewer false positives) and in 1 [21] the proportion was similar. In 2 comparisons [20, 22] the proportion of diagnoses missed by the CPR was greater, with a similar proportion classified as not having the disease. In 1 comparison [17] between the Oudega Rule and the combination of clinical judgment and Oudega Rule, the proportion of diagnoses missed was similar, with the rule classifying a larger proportion of participants as not having the disease (fewer false positives).

### Streptococcal throat infection

There were 3 studies of streptococcal throat infection.

In 2 comparisons [23, 24] between the Centor Score (Modified and Original score combined with Tomkins Management Rule) and 1 comparison between the Walsh score and clinical judgment [23] the proportion of diagnoses missed and the proportion of all participants classified as not having disease was similar. In these studies clinicians would likely have been aware that all study participants would have pharyngeal swabs taken for testing as per study protocol. This may lead to an overestimate of the proportion of participants classified as not having disease by clinical judgment.

### Foot and or ankle fracture

From 3 studies of foot and or ankle fracture, there were 3 (100%) comparisons between the Ottawa ankle and foot rules (OAR) and clinical judgment [26–28]. In all 3 comparisons the proportion of diagnoses missed by the CPR and clinical judgment was similar. In 1 of these [27] the rule classified a larger proportion of study participants as not having disease (fewer false positives) and in 2 comparisons [26, 28] the CPR classified a larger proportion of participants as having disease (more false positives). In the 2 comparisons from 2 studies [26, 28] in which the OAR classified a larger proportion of participants as having disease than clinical judgment, the clinicians when making a decision or diagnosis, would likely have been aware that all participants would be x-rayed as per study protocol [26] or would have known that an x-ray could be ordered at their discretion (28). This may lead to an overestimate of the proportion of study participants classified as not having disease by clinical judgment.

### Acute appendicitis

There were 2 studies of acute appendicitis.

In 1 comparison [29] between the Fenyo Score and the combination of score and clinical judgment, the proportion of diagnoses missed by the score was greater while the proportion classified as not having disease was similar. In 1 comparison [30] between the Modified Alvarado Score and clinical judgment, the proportion of diagnoses missed and the proportion of all study participants classified as not having disease was similar.

### Acute coronary syndrome, pneumonia, head injury in children, cervical spine injury, active pulmonary tuberculosis, malaria, bacteremia and influenza.

Of 8 studies (11 comparisons) addressing a variety of conditions, the CPRs showed either an improvement in the proportion of missed diagnosis or the proportion classified as not having disease, but this was often offset by a worsening of the other measure.

## Discussion

In this review, CPRs were rarely superior to clinical judgment and there was generally a trade-off between the proportion of study participants classified as not having disease and among those classified as not having disease, the proportion of missed diagnoses of disease. CPRs for the diagnosis of DVT generally classified a larger proportion of all participants as not having disease than clinical judgment, but this was often at the expense of missed diagnoses. In other disease areas, CPRs showed either an improvement in the proportion classified as not having disease or the proportion of missed diagnoses, but often with the trade-off of worsening the other measure. These findings, however, are limited by the small number of studies for many of the conditions, the design features and generally unclear or high risk of bias in many of the included studies.

Trade-offs in the proportion classified as not having disease and the proportion of missed diagnosis by CPRs and clinical judgment seen in this review probably represent differences in the diagnostic threshold for positivity of the two judgment methods. For example, CPRs might be developed to avoid missing people with disease and as such the threshold for positivity is set very low. The CPR would therefore likely be safer than clinical judgment where the threshold for positivity is implicitly set and variable between and within clinicians, but this is often at the expense of classifying fewer participants as not having disease (and thereby avoiding further testing or treatment). Whether clinical judgment or a CPR is the preferred judgment methods for a particular clinical condition will therefore depend on the relative benefits and harms arising from true positive and false positive diagnosis.

Variability in the proportion classified as not having disease and proportion of missed diagnoses of CPRs compared with clinical judgment, even amongst studies of the same CPR, may be explained in part by features of the clinical setting of the studies. Differences in study design and methodology, including the type of CPR tested (logistic regression model or other statistical technique), the rigour with which it was developed, the case-mix of the study population, ‘modifications’ to clinical judgment (with or without structured data collection), by whom (novice or experienced clinicians) or the way in which the result of the CPR is derived (calculation by clinician or researcher) may also explain the variation in performance seen in the studies included in this review. In many studies, clinicians collected diagnostic data on a structured data collection form. This systematic collection of diagnostic information may improve the observed diagnostic accuracy of the clinicians [39]. Clinician experience has also been shown to improve the accuracy of diagnosis [40].

Variability in the outcomes of clinical judgment and CPRs within conditions may also be explained by the method used to elicit clinical judgment, as the method used will likely be associated with the implicit threshold for positivity. In studies of appendicitis for example, clinical judgment was a clinician’s diagnosis of appendicitis or the clinician’s actual action to perform surgery or not. In studies of ankle fracture, clinical judgment was either a clinicians diagnosis of fracture or their intention to x-ray a patient, and for studies of sore throat, clinical judgment may have been a clinicians actual action to prescribe antibiotics or not or a clinicians statement of their intention to treat with antibiotics. The clinicians threshold for positivity will likely be higher for instance, if asked to provide a diagnosis (diagnostic threshold) than when asked of their intention to do further definitive testing (testing threshold). Where clinical judgment was elicited by obtaining a clinicians probability estimate on a continuous scale, there was also variation in the thresholds applied by study researchers. For studies of pulmonary embolism for example, thresholds were applied at probabilities of 15 or 20%.

The design of the studies included in this review allows comparison of the performance of CPRs and clinical judgment when applied independently. In practice, however CPRs are likely to be used as tools to support or complement clinical judgment. When used in this manner, the performance of the diagnostic CPRs may vary from that shown in this review. The effect of a CPR when used in conjunction with clinical judgment can only be fully tested in a study design in which participants are assigned (ideally randomly) to apply or receive clinical judgment alone or clinical judgment with access to a CPR. However, studies of diagnostic accuracy or incremental value [41, 42] provide a useful and less costly interim step in the evaluation of CPRs prior to a randomised controlled trial and can guide future research.

Our study shows that, in the context of medical diagnosis, CPRs do not consistently classify more individuals as not having disease or miss fewer diagnoses among those classified as not having disease than clinical judgment. This is in contrast to several reviews comparing clinical and statistical methods of prediction, often combining studies from fields as diverse as education, criminology and healthcare, which have generally found statistical methods to be superior [3–5]. A more recent body of research however has found that when formally tested, heuristics, proposed as models of human judgment are, in some situations as accurate as, or more accurate than statistical models [6]. A review comparing the diagnostic accuracy of doctors and statistical tools for acute appendicitis [43] found that statistical tools had greater specificity than clinicians. However, most of the studies included in this review were excluded from the present review because a) the statistical tools and clinical judgment were not applied at the same time point or b) the statistical tools and clinical judgment were not applied to the same participants.

Due to variation in the design and purpose of the included studies, we did not attempt meta-analysis across or within study conditions. Instead, we compare CPRs and clinical judgment using two measures 1) the proportion of all study participants classified as not having disease (a measure or efficiency) and 2) the proportion of participants among those classified as not having disease, who actually have the disease (false negative rate, a measure of safety). Because many CPRs seek to either improve diagnosis or identify a group of patients who do not require additional testing, we believe these are the most clinically relevant measures. Though these measures are dependent on the prevalence of the disease in the study population, the studies were judged to have been undertaken in relevant clinical settings. Traditional measures of diagnostic accuracy, such as sensitivity, specificity and area under a receiver operator characteristic curve are often favoured accuracy metrics because they are commonly believed to be unaffected by disease prevalence, though this has recently been shown not to be the case [44]. The proportion of participants classified as having disease and the proportion with false positive results can also be obtained from Figs 3 and 4 and the traditional measures of diagnostic accuracy from Tables 3 and 4.

The majority of included studies were judged to be at high or unclear risk of bias on 2 or more of the 4 risk of bias domains assessed. Differential verification (the results of clinical judgment or the CPR influence the performance of reference tests) and incorporation bias (the results of the CPR are used to make the final diagnosis) affected many studies, particularly studies of DVT and PE. Further, studies commonly did not include all eligible cases in the analysis and often it was not clear whether researchers applying a CPR retrospectively to a dataset were blind to the results of the reference standard. The design of studies of ankle fracture and streptococcal throat infection may also have led to inaccurate estimates of the diagnostic accuracy of clinical judgment. In these studies, the clinicians’ diagnosis or decision that x-ray or antibiotics are necessary may have been influenced by knowledge that all or most study participants would undergo confirmatory testing with an x-ray or throat swab. In this review, in two of the three studies of ankle and or foot fracture, the Ottawa Ankle Rules were considerably less efficient than clinical judgment that a fracture was present or that an x-ray was necessary. This finding conflicts with that a multicentre randomised controlled trial in which application of the rules lead to x-rays for 79% of study participants compared to 99.6% of participants when the decision was made by emergency department physicians [45].

The database searches to identify studies for the review were conducted up to March 2013 and eligible studies may have been published since this time. Because of the size of the search, not all titles and abstracts identified in electronic searches were screened by 2 reviewers. However, a second reviewer screened a subset of titles and did not find any additional studies. The search terms used may not have located all eligible studies, but manual searches of systematic reviews of CPRs and comprehensive reference and citation checking minimise this possibility. As assessment of the risk of bias in the studies deriving the CPRs or the ‘useability’ features of the CPRs evaluated in this review was not conducted, but updates to this review should seek to do this. Such information may assist in the interpretation of the results of the review.

While CPRs show promise as a way of improving clinical decision making, to date there have been limited studies comparing, in the same participants, the accuracy of CPRs and clinical judgment, and those studies often had design issues that raised the potential for bias and made interpretation of their results difficult. Though detailed guidance on the validation and evaluation of prediction models and rules is available [46, 47], guidance on issues specific to studies comparing the diagnostic performance of CPRs and clinical judgment may improve this situation. To inform of the potential of diagnostic CPRs to improve diagnosis and patient outcomes when the CPR is used in combination with clinical judgment, particularly in situations where the clinician has a high degree of uncertainty, an analysis of studies comparing care provided when clinicians have access to a diagnostic CPR with usual care would be useful.

## In Summary

The limited studies included in this review show that none of the CPRs evaluated to date are clearly superior to clinical judgment across a range of medical conditions. They also show variation in the comparative performance of clinical judgment and CPRs between studies for the same condition and between the same CPRs. There is generally a trade off in the proportion classified as not having disease and missed diagnosis that is most likely due to different thresholds for positivity associated with clinical judgment and CPRs. The current review highlights some of the methodological issues relating to the conduct of studies comparing CPRs and clinical judgment, with design features of many of the included studies increasing the potential for bias.

## Supporting Information

S1 Table Study protocol. (DOCX)

S2 Table PRISMA checklist. (DOCX)

S3 Table Electronic database search. (DOCX)
