# Supplementary material for: A Systematic Review of Studies Comparing Diagnostic Clinical Prediction Rules with Clinical Judgment
Source: PLoS One. 2015 Jun 3;10(6):e0128233. doi: 10.1371/journal.pone.0128233 (PMC4454557; doi:10.1371/journal.pone.0128233)
Supplement: S1 Table — (DOCX) [file pone.0128233.s001.docx]

**S1 Table -** Study protocol

| **Title** |
| --- |
| The comparative accuracy and added value of clinical prediction rules and clinicians for making diagnoses |
| **Reviewers** |
| Sharon Sanders, Jenny Doust, Paul Glasziou |
| **Background** |
| When used in the diagnostic context, prediction models, which are made up of a parsimonious combination of clinical characteristics, provide an estimate of the probability of disease presence. They are known as prediction or decision rules when cut-offs in probability are identified and used to produce clinically relevant risk categories (Steyerberg 2009). They are designed to reduce or make explicit diagnostic uncertainty, objectify, simplify, make more consistent and increase the accuracy of clinicians diagnosis.  However, the comparative accuracy of clinicians judgements, clinicians judgements aided by CPRs and CPRs is unclear. A large body of research dating back to the 1950s on the relative merits of clinical versus statistical or mechanical prediction has found that statistical predictions (based on empirically established relations between data and the condition of interest) consistently outperform clinical methods of prediction (clinician judgments based on informal or intuitive process). While the findings of this research remain unchallenged, its use to clinicians in the diagnostic process is limited for several reasons. Firstly, the numerous systematic and non-systematic reviews have, in the main, summarised findings from comparisons in fields as diverse as education, finance, health and criminology (Grove 2000, Dawes 1989). Consequently, there is limited specific information about the application of the findings to diagnostic tasks commonly encountered in medicine. Secondly, the most recent systematic review of studies comparing clinical and statistical prediction in the field of counselling psychology found that the size of the effect (which favoured the accuracy of statistical over clinical methods) varied according to type of prediction being made and type of statistical formula being used. Thirdly, in practice CPRs may be used in combination with varying degrees of clinician judgement. Whilst existing research in counselling psychology has also found that statistical prediction alone outperforms the clinician when using or having access to statistical methods (AEgisdottir 2006), how clinicians perform when using a CPR (partially or fully, correctly or incorrectly) in comparison to their performance when not using a CPR is unknown.  Guides for developing clinical prediction models state the importance of comparing clinician predictions with those of prediction models during validation or impact assessment, but preferably during development of the prediction model (Reilly 2006, Toll 2008, Brehaut 2005). If clinicians predictions of probabilities are shown to outperform the probabilities provided by a prediction model, it is unlikely the model will be used in practice. Conversely demonstration that the model alone or in combination with clinician judgement performs better than the clinician alone may assist in the acceptance and implementation of the rule. |
| **Objectives** |
| This systematic review will provide up to date and specific information on the comparative accuracy of validated current diagnostic clinical prediction rules and clinician judgement aided or unaided by clinical prediction rules in the field of medicine. Due to difficulties with objectively defining the presence of disease, a decision was made to exclude studies in the fields of psychology and psychiatry from this review.  **What is the comparative accuracy and added value of clinical prediction rules and clinician judgement for making diagnoses in people with suspected disease?** |
| **Methods**  *Inclusion and exclusion criteria*  Studies comparing the accuracy of a clinicians estimation of disease probability, diagnosis or decision on further testing or treatment with a clinicians estimate aided by a CPR or a CPR alone and including a current reference standard test will be included. Inclusion and exclusion criteria are tabulated.   \| Inclusion \| Exclusion \| \| --- \| --- \| \| Study design \| \| \| - Cohort or cross sectional studies whose objective or intention is to compare, or who present data for the comparison between a clinicians estimate, clinicians estimate combined with a CPR or with a CPR. A ‘clinicians estimate’ may be a statement of probability or diagnosis or need for further testing or may also be a proxy for diagnosis (ie decision to perform surgery). - Study includes a currently acceptable reference standard test. - Studies with multivariable modelling to determine the incremental value of the CPR over and above clinician judgement - Studies relevant to the field of medicine \| - The study does not include a reference standard test or what is considered to be a current and adequate reference standard test or compares either CPR or clinician judgement alone to a reference standard - Studies predominantly relevant to psychology or psychiatry - Studies in which the CPR and clinician estimates are performed in different cases - Studies in which a selected sample is used (ie only those having surgery) - Studies in which ‘clinicians estimate’ is normal management (ie decision to perform surgery) that do not do adequately followup cases negative by clinician or indicate a priori the intention to follow up negative cases (ie studies in appendicitis that do not perform post discharge followup of cases considered negative by the clinician) or that compare a CPR based on data collected at a different time point to the data used to inform the clinicians estimate or normal management (ie if data entered into the CPR is collected at admission versus the decision of the clinician to perform surgery at a later time point after further examination, testing and consultation) \| \| Index test 1 (clinician judgment alone) \| \| \| - a clinician of any type provides a probability of disease, diagnosis or makes a decision on further testing or management without formally using a CPR \|  \| \| Index test 2 (CPR alone or clinician judgement aided by CPR) \| \| \| - a clinician of any type provides a probability of disease, diagnosis or makes a decision on further testing or management based on a combination of clinical judgement and use of a CPR - The CPR is a combination of findings from history, physical examination or diagnostic tests that are statistically meaningful predictors of a condition of interest. CPRs developed using the following methods will be included; univariate analysis, Bayesian analysis, multivariable analysis, neural networks, CART analysis and presented as scoring systems, nomograms, regression equations, flow diagrams and can be paper based or electronic based. - Diagnostic CPRs are to be included. Diagnostic CPRs are considered to be those that determine the probability of a condition of interest, make a diagnosis or assist in deciding when further evaluation is likely to be helpful. The CPR may be part of a multifaceted diagnostic approach or strategy - The CPR is applied to actual patients - The CPR is considered to be current, that is relevant to and useable in current practice - Studies comparing clinician judgement to an already validated CPR or a CPR that has been derived in a sample other than the one in the potentially relevant study will be included. Studies comparing clinician judgement to a CPR that has been temporally validated will also be included - The CPR is used by the clinicians themselves (data collected and or result determined), information derived from the CPR is provided to the clinician or the CPR is applied retrospectively to the data independently of the clinician \| - Decision aids/tools or algorithms developed based on expert opinion or current ‘best’ practice - The CPR is used to interpret results of clinical tests i.e. ECG, X-ray - The CPR is applied by students or patients - The CPR is prognostic or interventional - The CPR is designed to assist in determining the severity of disease or making diagnosis across multiple disease groups - The CPR has been derived or internally validated when compared to the clinician \| \| Outcome \| \| \| - Study provides data from which sensitivity and specificity can be calculated. - Study provides data on the incremental value of the CPR above clinician judgement \| - Studies reporting only crude accuracies excluded - CPR or clinician data is missing for >=20% of cases \|   *Search strategy*  Potentially relevant studies will be identified though electronic searches of the following databases (from inception); MEDLINE (via Ovid), EMBASE and CINAHL. Sensitive search strings using free text and thesaurus terms combined with Boolean operators will be developed for each database. No language restrictions or study design filters will be used. PubMed Clinical Queries will be used to search for systematic reviews of diagnostic clinical prediction rules and studies included in the reviews retrieved and checked against the reviews inclusion criteria. To identify additional published, unpublished and ongoing studies, the reference lists of relevant studies will be checked and included studies forward searched using Scopus and the Science Citation Index Expanded in Web of Science (via Web of Knowledge). In addition, related citations will be checked using PubMed’s Related Citations link and keyword searches of Google Scholar undertaken. Titles and abstracts identified in the searches will be imported into endnote and de-duplicated.  *Study selection process*  Titles and abstracts identified in the searches will undergo an initial screen by one reviewer and obviously irrelevant articles excluded. Titles and abstracts will be screened in Endnote and sorted into groups (potential inclusion or exclusion). To assess the reliability of this process a second person (RT) will independently screen a sample (15%) of the titles and abstracts. Studies identified as being potentially relevant by SS will be obtained in full text and independently assessed by two reviewers (SS, JD) against the review inclusion criteria. A third reviewer (PG) will be available to resolve discrepancies in the selection process.  *Risk of bias assessment*  Included studies will be independently assessed for risk of bias by JD and SS using QUADAS-2. The standard QUADAS-2 tool will be piloted and adjusted if necessary. Discrepancies between reviewers will be discussed and if unresolved opinion will be sought from third reviewer (PG).  *Data extraction strategy*  A data collection form will be developed and piloted. Data from each eligible study will be independently extracted by two reviewers (SS, JD). The following data will be extracted from study reports; the target condition, number with the target condition, the reference standard test and definitions used, study setting, participants to whom clinical judgment and CPR being applied, how the CPR is applied and how clinicians make their clinical judgment. Study results to be extracted include thresholds for CPR and clinical judgment, data for 2x2 tables for CPR and clinical judgment and AUROC and sensitivity and specificity as calculated by study authors if provided. |
