# Supplementary material for: A Systematic Review of Studies Comparing Diagnostic Clinical Prediction Rules with Clinical Judgment
Source: PLoS One. 2015 Jun 3;10(6):e0128233. doi: 10.1371/journal.pone.0128233 (PMC4454557; doi:10.1371/journal.pone.0128233)
Supplement: S3 Table — (DOCX) [file pone.0128233.s003.docx]

**S3 Table -** Electronic database search strategies

| MEDLINE was searched using the Ovid  interface on 24/4/13 for the period 1946 to  March Week 4 2013 | Embase was searched using embase.com on 27/2/12 for the period 1974 to January 2012 | Cumulative Index to Nursing and Allied Health Literature (CINAHL) using the EBSCOhost interface on 27/2/12 for the period 1982 to January 2012 | PubMed was searched on 28/4/13 for systematic reviews of clinical prediction rules |
| --- | --- | --- | --- |
| 1. ((clinician* or professional* or practitioner* or physician* or nurse*) adj3 (judgment* or judgement*or estimate* or diagno* or prediction* or assess* or decision* or intuition* or impression* or evaluation* or probabilit* or empirical* or subjectiv* or implicit* or unaided or unstructured or accuracy or performance)).ti,ab.  2. (clinical adj3 (judgment* or judgement* or estimate* or diagnos* or assessment* or impression* or probabilit*)).ti,ab.  3. ((empirical or subjective or implicit or unaided or unstructured) adj3 (judgment* or judgement*or estimate* or diagnos* or prediction* or assessment* or decision* or impression* or evaluation* or probabilit*)).ti,ab.  4. 1 or 2 or 3  5. *Decision Support Techniques/  6. (scor* or rule* or model* or guide* or algorithm* or protocol* or "formal estimate" or "formal estimates").ti.  7. 5 or 6  8. 4 and 7 | 1. score*:ti OR rule*:ti OR model*:ti OR guide*:ti OR algorithm:ti OR protocol*:ti  2. (clinician* OR professional* OR practitioner* OR physician*) NEAR/3 (judgment OR judgement OR estimate OR diagnosis OR prediction OR assessment OR decision OR intuition OR impression OR evaluation OR probability OR empirical OR subjective OR implicit OR unaided OR unstructured)  3.(empirical OR subjective OR implicit OR unaided) NEAR/3 (judgment OR judgement OR estimate OR diagnosis OR prediction OR assessment OR decision OR impression OR evaluation OR probability)  4. 2 or 3  5. 1 and 3 | 1. 1. TI (score* OR rule* OR model* OR guide* OR algorithm OR protocol OR "formal estimate") 2. 2. TI ( ((clinician* OR clinical OR professional* OR practitioner* OR physician* OR nurse*) N3 (judgment OR estimate OR diagnosis OR prediction OR assessment OR decision OR intuition OR impression OR evaluation OR probability OR empirical OR subjective OR implicit OR unaided)) ) OR AB ( ((clinician* OR clinical OR professional* OR practitioner* OR physician* OR nurse*) N3 (judgement OR judgment OR estimate OR diagnosis OR prediction OR assessment OR decision OR intuition OR impression OR evaluation OR probability OR empirical OR subjective OR implicit OR unaided)) ) 3. 3. TI ( ((clinical) N3 (judgment OR estimate OR diagnosis OR assessment OR impression OR probability)) ) OR AB ( ((clinical) N3 (judgment OR estimate OR diagnosis OR assessment OR impression OR probability)) ) 4. 4. TI ( ((empirical OR subjective OR implicit OR unaided) N3 (judgment OR judgement OR estimate OR diagnosis OR prediction OR assessment OR decision OR impression OR evaluation OR probability)) ) OR ( ((empirical OR subjective OR implicit OR unaided) N3 (judgment OR estimate OR diagnosis OR prediction OR assessment OR decision OR impression OR evaluation OR probability)) ) 5. 5. 2 or 3 or 4 6. 6. 1 and 5 | 1. 1. Medline[tiab] OR (systematic[tiab] AND review[tiab]) OR meta-analysis[ptyp] 2. 2. Score[ti] OR scores[ti] OR rule[ti] OR rules[ti] 3. 3. 1 AND 2 |
